# Capture‐based next‐generation sequencing reveals multiple actionable mutations in cancer patients failed in traditional testing

**DOI:** 10.1002/mgg3.201

**Published:** 2016-01-10

**Authors:** Jing Xie, Xiongxiong Lu, Xue Wu, Xiaoyi Lin, Chao Zhang, Xiaofang Huang, Zhili Chang, Xinjing Wang, Chenlei Wen, Xiaomei Tang, Minmin Shi, Qian Zhan, Hao Chen, Xiaxing Deng, Chenghong Peng, Hongwei Li, Yuan Fang, Yang Shao, Baiyong Shen

**Affiliations:** ^1^ Research Institute of Pancreatic DiseaseRuijin HospitalSchool of MedicineShanghai Jiao Tong UniversityShanghaiChina; ^2^ Department of PathologyRuijin HospitalSchool of MedicineShanghai Jiao Tong UniversityShanghaiChina; ^3^ Pancreatic Disease CentreRuijin HospitalSchool of MedicineShanghai Jiao Tong UniversityShanghaiChina; ^4^ Department of Research and DevelopmentGeneseeq Technology Inc.TorontoOntarioCanada; ^5^ Department of Laboratory MedicineRuijin HospitalSchool of MedicineShanghai Jiao Tong UniversityShanghaiChina; ^6^ Shanghai Institute of Digestive SurgeryRuijin HospitalSchool of MedicineShanghai Jiao Tong UniversityShanghaiChina; ^7^ Department of Medical BiophysicsUniversity of TorontoTorontoOntarioCanada

**Keywords:** Next‐generation sequencing, molecular diagnosis, cancer panel, targeted therapy

## Abstract

**Background:**

Targeted therapies including monoclonal antibodies and small molecule inhibitors have dramatically changed the treatment of cancer over past 10 years. Their therapeutic advantages are more tumor specific and with less side effects. For precisely tailoring available targeted therapies to each individual or a subset of cancer patients, next‐generation sequencing (NGS) has been utilized as a promising diagnosis tool with its advantages of accuracy, sensitivity, and high throughput.

**Methods:**

We developed and validated a NGS‐based cancer genomic diagnosis targeting 115 prognosis and therapeutics relevant genes on multiple specimen including blood, tumor tissue, and body fluid from 10 patients with different cancer types. The sequencing data was then analyzed by the clinical‐applicable analytical pipelines developed in house.

**Results:**

We have assessed analytical sensitivity, specificity, and accuracy of the NGS‐based molecular diagnosis. Also, our developed analytical pipelines were capable of detecting base substitutions, indels, and gene copy number variations (CNVs). For instance, several actionable mutations of *EGFR*,*PIK3CA*,*TP53*, and *KRAS* have been detected for indicating drug susceptibility and resistance in the cases of lung cancer.

**Conclusion:**

Our study has shown that NGS‐based molecular diagnosis is more sensitive and comprehensive to detect genomic alterations in cancer, and supports a direct clinical use for guiding targeted therapy.

## Introduction

The greatly improved understanding of molecular etiology of cancer (Chin and Gray 2008; Stratton et al. 2009; International Cancer Genome C et al., 2010; Mardis 2012) has changed systemic cancer treatment by using molecularly targeted drugs prescribed to an individual patient. Targeted therapies that are intended to be safer and more efficacious block the growth and spread of cancer by interfering with the molecules that are involved in growth, progression, and metastasis. Many targeted cancer therapies have been approved by the Food and Drug Administration (FDA) to treat specific types of cancer, such as Trastuzumab (Herceptin) in *ERBB2* (also known as *HER2*; OMIM*164870)‐amplified breast cancer, Imatinib (Gleevec) in BCR‐ABL (OMIM*151410; *189980) fusion‐positive chronic myelogenous leukemia (CML), Erlotinib (Tarceva) in *EGFR* (OMIM*131500)mutated non‐small‐cell lung cancer (NSCLC), and Vemurafenib (Zelboraf) in BRAF‐V600E (OMIM*164757) mutant melanoma (Stegmeier et al. 2010). More personalized cancer therapy will be achieved as there are now thousands of compounds in preclinical testing and clinical trials targeting hundreds of genomic alterations in cancer‐related genes involving innumerous cellular pathways (Barretina et al. 2012; Garnett et al. 2012). Moreover, certain somatic mutations can also impact the sensitivity or resistance to specific cancer therapies (Diaz et al. 2012; Camidge et al. 2014). In order to precisely match each individual or a subset of cancer patients with available targeted therapies, comprehensive molecular diagnosis tests need to be developed to characterize the genomic alterations occurring within individual tumors. Several technologies, including PCR, Sanger sequencing, mass spectrometric genotyping, fluorescence in situ hybridization (FISH), and immunohistochemistry (IHC) (Thomas et al. 2007; MacConaill et al. 2009; Dias‐Santagata et al. 2010; Ross 2011; McCourt et al. 2013), are currently in clinical use for the molecular assessment. However, due to technical limitations, none of these methodologies can be scaled to address the increasing number and variety of therapeutically relevant genomic alterations that occur across hundreds of cancer‐related genes (Cancer Genome Atlas N, 2012; Cancer Genome Atlas Research N, 2012; Nik‐Zainal et al. 2012a,b; Stephens et al. 2012).

Next‐generation sequencing (NGS), also known as massively parallel sequencing, is therefore becoming an attractive clinical diagnostic tool since it is able to accurately detect most genomic alterations in a single assay (Roychowdhury et al. 2011; Liang et al. 2012; Craig et al. 2013; Frampton et al. 2013). However, the clinical practice of this technology as a routine diagnostic test is still challenging. Firstly, the majority of cancer specimens are formalin‐fixed, paraffin‐embedded (FFPE), a process can damage DNA in different extends depending on the pathology processing protocol and the age of the sample (Hadd et al. 2013). Therefore, robust DNA extraction and sequencing library construction protocols need be standardized to improve the NGS data quality of FFPE samples. Secondly, many samples available for testing are small amount of material obtained from biopsies, which require optimized protocols that accommodate limited amount of DNA input (Kerick et al. 2011). Thirdly, some clinical specimens present low tumor content, which will influence the sensitivity of detection. As a result, uniformly high sequence coverage across all regions of interest and appropriate analysis algorithms are required.

In this study, we have developed and validated a NGS‐based cancer genomic diagnosis test targeting 115 cancer‐related and therapeutically relevant genes on multiple types of cancer and specimens. We have assessed the analytical sensitivity, specificity, and accuracy of the assay. We also developed NGS bioinformatics analysis pipeline for detecting base substitutions,indels, and gene copy number variations (CNVs), which can be efficiently validated by Sanger sequencing or real‐time quantitative PCR **(**qPCR) method. Our study showed that NGS‐based molecular diagnosis test is more sensitive in detecting genomic alterations in cancer, and supported a direct clinical use for this method to guide targeted therapy.

## Materials and Methods

### Ethical compliance

The patient information and clinical samples were obtained from the Ruijin Hospital. The sample collection and preparation protocol was approved by the Ruijin Hospital Ethics Committee (reference number: 2013‐70).

### DNA extraction

Four to eight 5–10 *μ*m FFPE sections were obtained per case. FFPE sections were then scraped into microcentrifuge tubes. The tissues were deparaffinized with 1 mL xylene at 56°C for 10 min, washed with 1 mL 100% ethanol for 5 min at RT, and then dried at 37°C for 10 min. QIAamp DSP DNA FFPE tissue kit (Qiagen, Valencia, CA, USA) was used to extract the genomic DNA from FFPE samples and DNeasy Blood & Tissue kit (Qiagen) was used to extract genomic DNA from blood and body fluid with in‐house modifications. DNA concentration was determined by Qubit dsDNA HS assay kit on the Qubit Fluorometer according to the manufacturing protocol (Life Technologies, Carlsbad, CA, USA). DNA quality (A260/280 and A260/230) was measured by Nanodrop‐2000 (Thermo Fisher Scientific, Waltham, MA, USA).

### Library preparation

Sequencing library was prepared by Illumina TruSeq DNA PCR‐Free Sample Preparation Kit (Illumina, San Diego, CA, USA) according to the manufacturing protocol. In brief, genomic DNA sample was fragmented into 350 or 550 bp in AFA fiber snap‐cap microTUBE using Covaris M220 (Covaris, Woburn, MA, USA). End repair and size selection were performed according to the fragment size, followed by 3′ end adenylation. Finally, multiple indexing adapters were ligated to the ends of the DNA fragments. Library concentration was determined using Qubit according to the manufacturing protocol. For low DNA input samples, PCR‐free library was further amplified with Illumina p5 (AATGATACGGCGACCACCGA) and p7 (CAAGCAGAAGA‐CGGCATACGA) primers in NEB Next High‐Fidelity 2XPCR Master Mix (NEB, Ipswich, MA, USA).

### Hybrid capture and sequencing

Different libraries with unique indexes were pooled together with desirable ratio to up to 2 *μ*g of total library input. A quantity of 5 *μ*g human cot‐1 DNA (Life Technologies) and 1 nmol of each xGen Universal blocking oligos (p5 or p7; IDT, Coralville, IA, USA) were added as blocking reagents. TruSight Cancer Panel Probes (Illumina) and customized xGen lockdown probes (IDT) were used for targeted enrichment, which collectively targets 115 cancer‐related genes (Table S1). A quantity of 10 *μ*L 2 × Hybridization buffer (0.5 mol/L Sodium phosphate buffer, pH 7.0, 1% SDS, 2 mmol/L EDTA, 2 × SSC and 4 × Denhardt's solution) was added to make the total reaction volume of 20 *μ*L. The hybridization mix was denatured on a thermal cycler at 95°C for 5 min, and then incubated 30 cycles of 1 min duration, starting at 94°C, then decreasing 1°C per cycle with final incubation at 65°C for 16–24 h. Dynabeads M‐270(50 *μ*L); (Life Technologies) was washed with Bind and Wash buffer (10 mmol/L Tris‐HCl, pH7.5, 2 mol/L NaCl, 1 mmol/L EDTA and 0.1% Tween‐20). Hybridization reaction was added to Dynabeads M‐270, and incubated for 30 min at RT with rotation. Beads were then washed at 65°C with Wash buffer I (1 × SSC/0.1% SDS) for 5 min, Wash buffer II (0.1 × SSC/0.1% SDS) for 5 min twice, and at RT with Wash buffer II for 5 min, and finally, Wash buffer III (0.2 × SSC) for 30 sec. Captured libraries were eluted from beads by boiling beads in DNase/RNase‐free water at 98°C for 10 min, followed by postcapture amplification with Illumina p5 and p7 primers in NEB Next High‐Fidelity 2 × PCR Master Mix. Postcapture amplified library was purified and quantified by qPCR using KAPA Library Quantification kit (KAPA Biosystems, Boston, MA, USA). Library fragment size was determined by Agilent Technologies 2100 Bioanalyzer using a High Sensitivity DNA chip (Agilent Technologies, Santa Clara, CA, USA). Capture‐enriched library was sequenced on Illumina MiSeq NGS platform (Illumina) according to its instruction.

### Sequence data processing

Trimmomatic (Bolger et al. 2014) was used for FASTQ file quality control. Leading/trailing low quality (below quality 15) or N bases were removed. Reads from each sample were mapped to reference sequence hg19 (Human Genome version 19) using Burrows–Wheeler Aligner (BWA) (Li and Durbin 2009) with modified parameters. SNPs/indels were identified using modified Haplotype Caller in Genome Analysis Toolkit (GATK) (DePristo et al. 2011). Enrichment efficiency was determined based on the percentage of reads that map to the targeted regions with 150 bp padding. (CNVs) were detected using ADTEx (Amarasinghe et al. 2013) with default parameters. In brief, CNVs were identified using tested sample and normal human hapmap DNA NA18535 average read depths at each captured region (exonic region). Proposed discrete wavelet transform (DWT) was used to reduce intrinsic noise. The copy number gains/losses of each targeted region are performed by a Hidden Markov Model (HMM).

### Validation of SNPs/Indels and CNVs

SNPs/Indels were validated by Sanger sequencing. 200–500 bp of targeted DNA area was amplified by PCR using 2× AccuStart^TM^ II PCR SuperMix (Quanta BioSciences, Gaithersburg, MD, USA). PCR products were purified and sequenced by ABI 3730xl DNA Analyzer.

For CNVs validation, qPCR primers were designed for targeted exons of test genes and ZNF80 (reference gene) by Primer‐blast (Table S2). Normal human hapmap genomic DNA NA18535 was used as normal control sample. qPCR reactions was performed in triplicates using 2× SYBR Select Master Mix (Life Technology).

## Results

### Extraction of DNA from clinical specimens

In this study, we chose 14 samples from 10 different patients with different specimens and cancer types (Table 1). These samples included blood, tumor FFPE, and body fluid from the patients of lung cancer, colon cancer, rectal cancer, breast cancer, and neuroectodermal tumor crossing different genders and age ranges. Four cancer patients (patient 7–10) had both tumor/body fluid and matching blood samples.

**Table 1:** Patient sample information

Patient ID	Sample ID	Gender	Age at test	Type of cancer	Sample type
1	F1311260008	Female	64	Lung cancer, SCC	FFPE
2	B1312160009	Female	61	Colon cancer	Blood
3	F1312230017	Male	54	Lung cancer, SCC	FFPE
4	F1401170002	Male	57	Rectal cancer	FFPE
5	F1401170004	Male	53	Lung adenocarcinoma	FFPE
6	F1402240017	Male	20	Neuroectodermal tumor	FFPE
7	F1410200833 B1410200832	Female	52	Lung adenocarcinoma	FFPE
8	C1409280774 B1409280773	Male	67	Lung adenocarcinoma	Pleural fluid
9	F1411100940 B1411100938	Female	53	Breast cancer	FFPE
10	F1412241251 B1412241250	Female	66	Lung adenocarcinoma	FFPE

SCC, squamous cell carcinoma; FFPE, formalin‐fixed, paraffin‐embedded.

Genomic DNA from blood samples or body fluid was extracted using DNeasy Blood & Tissue kit with good quantity and quality. However, DNA extraction from FFPE samples remains a challenge. To optimize the extraction condition, we have explored several different methods and established the protocol using xylene for deparaffinization followed by extracting DNA with QIAamp DSP DNA FFPE tissue kit. In general, genomic DNA extracted from FFPE samples had certain level of fragmentation. As shown in Figure S1, FFPE DNA sample 1–2 showed a moderate fragmentation, while sample 3 showed a severe fragmentation with DNA fragments ranging from 100 to 1000 bp. We also observed that DNA quantification with Qubit dsDNA HS assay was more accurate compared with Nanodrop analysis. Using our optimized extraction protocol, the FFPE‐derived tumor DNA samples were all of quantity and quality sufficient for constructing NGS libraries on Illumina sequencing platform.

### Targeted next‐generation sequencing for cancer‐related genes

**Figure 1:**
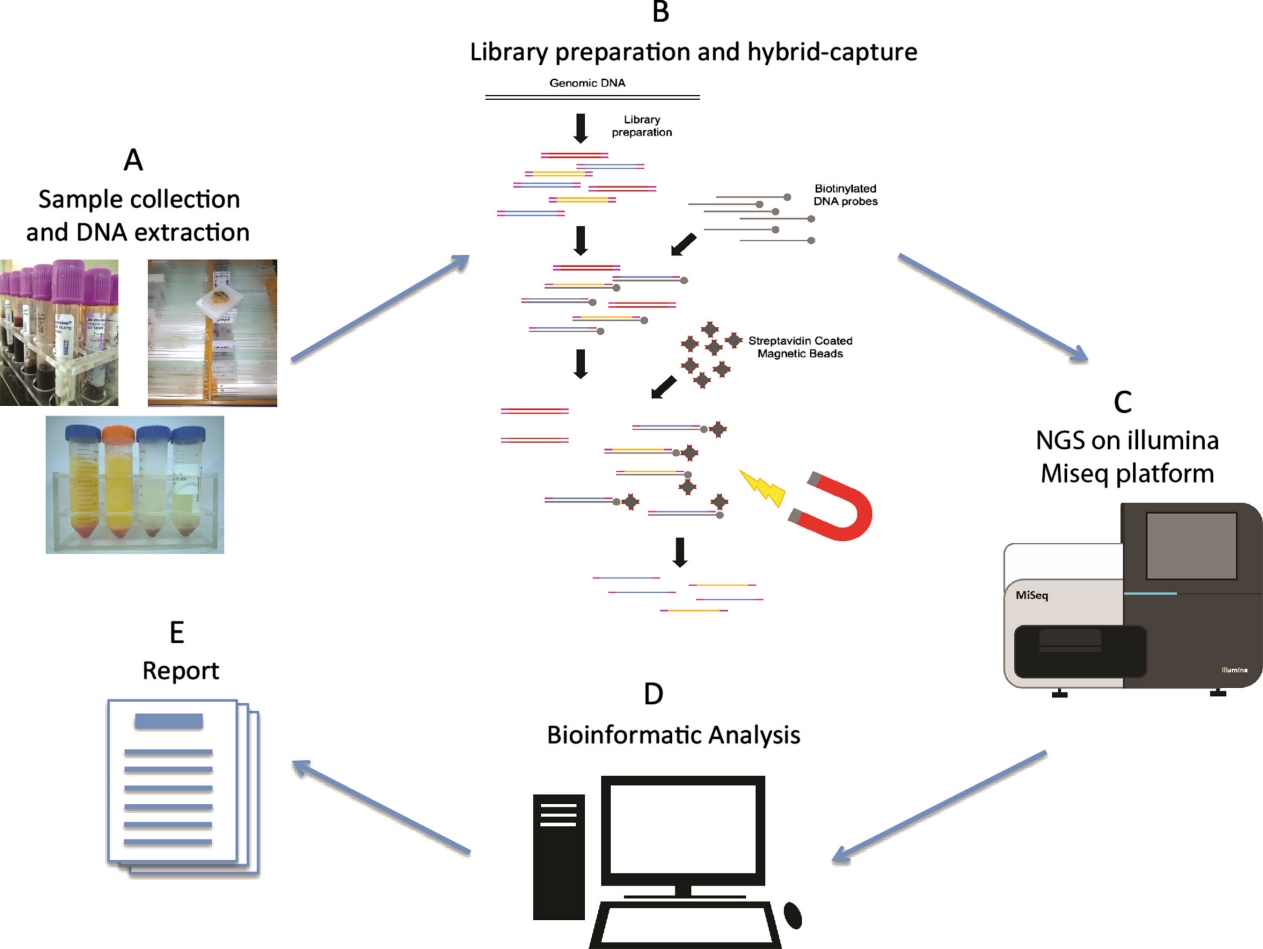
Targeted next‐generation sequencingNGS‐based cancer genomic testing workflow. (A) Clinical formalin‐fixed, paraffin‐embeddedFFPE biopsy/surgical specimens or blood samples were collected. Genomic DNA was extracted using different method according to the sample types. (B) Whole‐genome sequencing library for Illumina platform was prepared. Indexed sequencing adaptors were added to the libraries, and libraries were pooled accordingly. Regions/genes of interest were target enriched by hybridization with biotin‐labeled DNA probes and then captured by streptavidin magnetic beads. Enriched libraries were further amplified for sequencing. (C) Libraries were sequenced on Illumina Miseq platform. (D) Sequencing data was analyzed through a customized bioinformatic pipeline designed to detect SNVs, indels, and copy number variationsCNVs. (E) Detected mutations were interpreted according to clinical significance and reported.

In this study, we have developed an NGS‐based cancer genomic diagnostic test (Fig. 1) targeting 115 cancer‐related and therapeutically relevant genes (Table S1). Fragmented DNA underwent whole‐genome sequencing library construction. Size distribution of the constructed libraries was analyzed by Agilent Bioanalyzer (Fig. S2A). We note that the average library insert size of FFPE genomic DNA was smaller than blood counterparts, due to the fragmented nature of FFPE DNA. Regions or genes of interest were then capture enriched by biotin‐labeled DNA probes through hybridization, and amplified postenrichment. Using the Illumina Miseq platform, the hybrid‐capture‐enriched libraries were sequenced to high uniform depth. Library preparation and target enrichment protocols have been optimized to assure even coverage, low PCR duplicates, and robust performance for different type of samples.

Sequencing data was analyzed by our self‐developed bioinformatics analysis pipeline for accurately detecting multiple classes of genomic alterations, including base substitutions (SNVs), indels, and gene (CNVs) (See Materials and Methods). The target capture and sequencing performance were summarized in Table 2. Blood samples were sequenced at 50–100× mean coverage, while tumor samples were sequenced up to 200–500× mean coverage depending on its tumor content within the samples in order to identify low abundance gene mutations. With our optimized DNA extraction and library preparation protocols for the poor quantity or quality samples, the uniformity of coverage at regions of interest (percentage of coverage >0.2× mean coverage) was able to reach 93% for all types of samples together with significantly reduced PCR duplicates. Our protocol dramatically improved the coverage depth with the similar amount of sequencing data, resulting in >92% of target bases being spanned by at least 50 sequencing reads for tumor samples. The on‐target rate of all the sequencing reads was able to reach 75–88% on our 115 genes target panel.

**Table 2:** Target capture and sequencing performance

Sample ID	Total aligned reads	Read length (bp)	Mean coverage	Uniformity of coverage, % (Pct > 0.2*mean)	Target coverage at 10× (%)	Target coverage at 20× (%)	Target coverage at 50× (%)	On‐target rate (%)
F1311260008	1099120	150	62×	61.67	65.43	53.03	35.48	65.61
B1312160009	478099	250	47×	71.87	70.64	54.37	30.38	63.81
F1312230017	809335	250	65×	72.73	76.97	65.59	39.45	67.4
F1401170002	7210039	75	76×	80.73	85.78	76.01	51.58	69.89
F1401170004	8116131	75	94×	75.04%	84.49	73.81	50.24	72.13
F1402240017	3737573	250	389×	70.96	88.51	85.19	76.76	71.45
F1410200833	4183073	150	488×	92.71	93.77	93.65	93.6	85.63
B1410200832	2380225	150	274×	93.23	93.79	93.68	93.37	82.26
C1409280774	3741776	250	354×	92.91	93.88	93.74	93.40	88.87
B1409280773	339595	250	57×	93	93.28	90.61	57.02	79.18
F1411100940	1108491	250	243×	92.66	93.60	93.54	92.54	86.87
B1411100938	452129	250	87×	93.14	93.65	92.80	82.27	80.24
F1412241251	1766176	300	260×	92.14	93.68	93.54	92.20	87.17
B1412241250	340075	300	68×	92.43	93.03	90.41	66.75	79.79

### Identification and validation of SNP and indels in targeted genes

**Figure 2:**
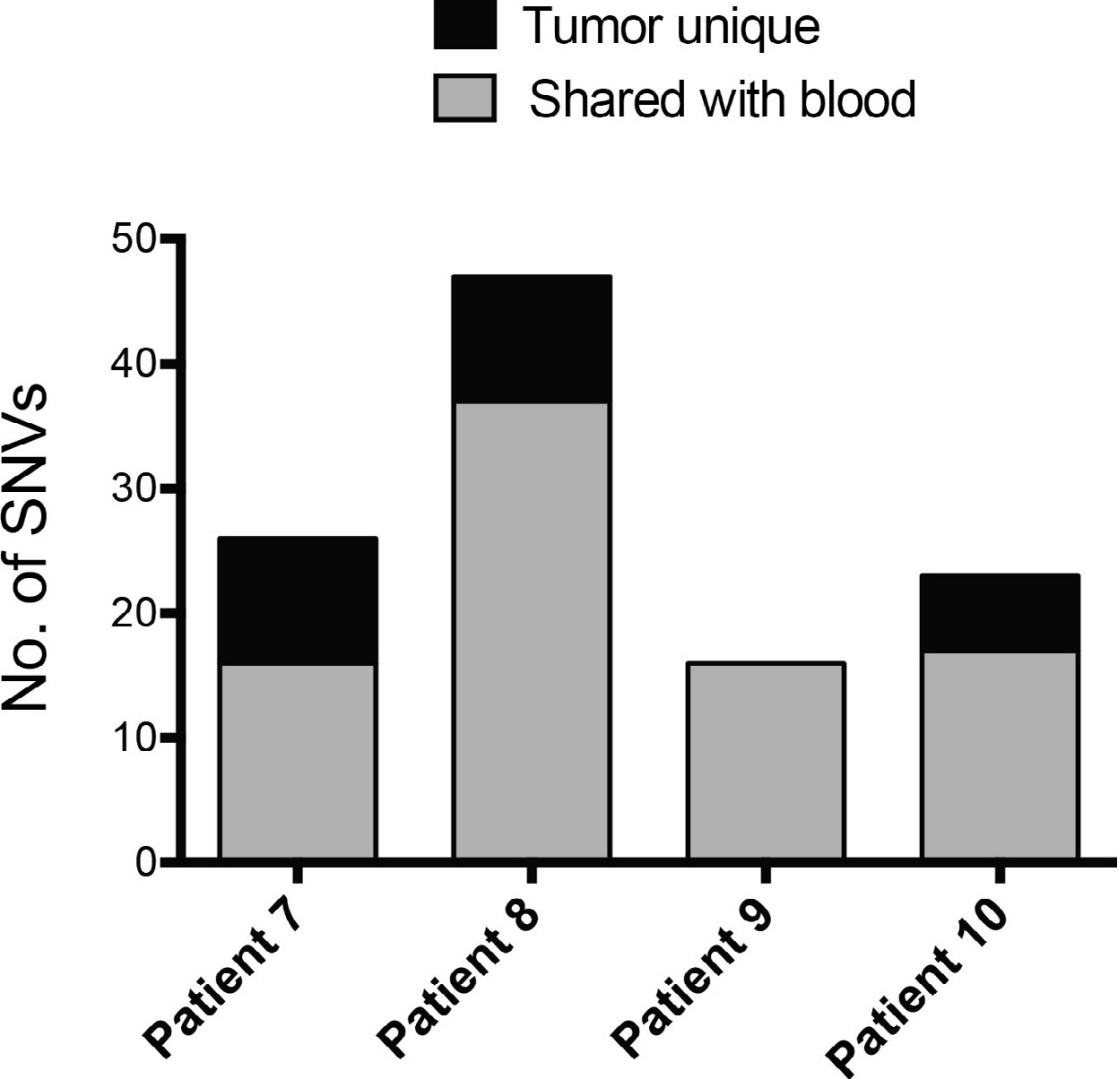
Overlap of SNP calls between tumor samples and matching blood samples at positions without dbSNP variants.

**Figure 3:**
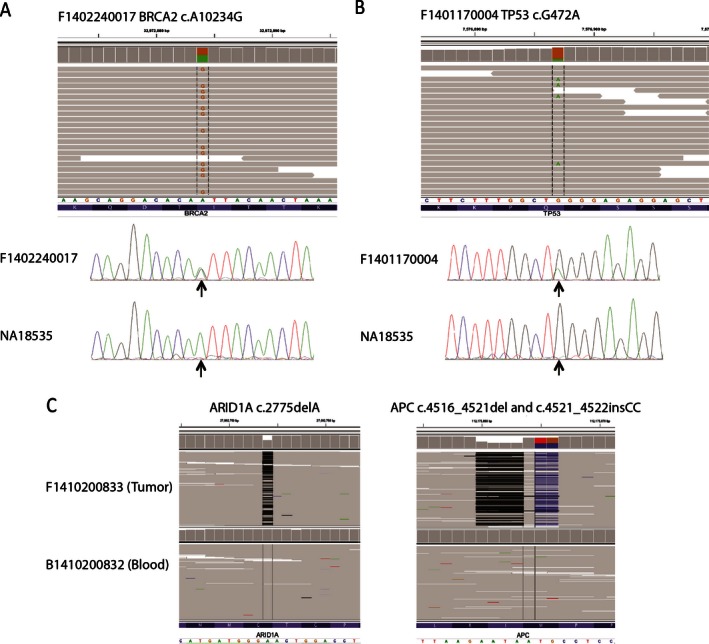
Examples of SNV and indels detected by next‐generation sequencing (NGS). Sequence alignment data was viewed by Integrative Genomics Viewer (IGV) (A and B top panel, and C).The two vertical discontinued lines framed the aligned bases at the variant site. A coverage track as a gray bar chart for each locus was shown on top of the alignment track. If a nucleotide differs from the reference sequence in greater than 10% of quality‐weighted reads, Integrative Genomics ViewerIGV colors the bar in proportion to the read count of each base. In alignment track, read bases that match to the reference sequence are displayed in gray. Read bases that do not match the reference are labeled and color coded. Base color code: green for A; blue for C; orange for G; red for T). RefSeq Gene track was shown at the bottom. Sanger sequencing validation for SNVs in the tumor sample and normal control NA18353 DNA were shown at the bottom in A and B. Black arrow indicated the mutant site tested.

SNPs and indels were identified using Haplotype Caller in GATK (DePristo et al. 2011). The known germline variants captured in dbSNP and in the 1000 genomes project were removed from all the SNPs called, thus showing the list of private germline variants and somatic mutations. By comparing with tumor‐matching blood sample control, the private germline variants were further removed from somatic mutations (Fig. 2). The examples for SNPs and indels detected by NGS were shown in Figure 3. To validate the accuracy of base substitution/indels detection, Sanger sequencing was used for validating the mutations contained in the PCR amplified DNA fragments. In total, 28 base substitution/indels detected by NGS pipeline were tested (Table S3 and Fig. 3), within which 27 could be confirmed by Sanger validation. Studies have shown that the value of the mutant allele frequency, beyond which it is not detectable with confidence, is around 15% for the point mutations, and around 10% for the frame shift mutation (Chen et al. 2014). The mutation *RECQL4* (c.448T>A), which could not be validated by Sanger sequencing, had a low frequency as 9% (46 out of 511 reads), suggesting that Sanger sequencing is less capable of detecting low‐frequency mutation. The cut‐off value of mutant frequency to be reported was set at 5% for tumor samples and 10% for blood samples, with at least 5 reads for mutant allele.

### Identification and validation of CNV in targeted genes

**Figure 4:**
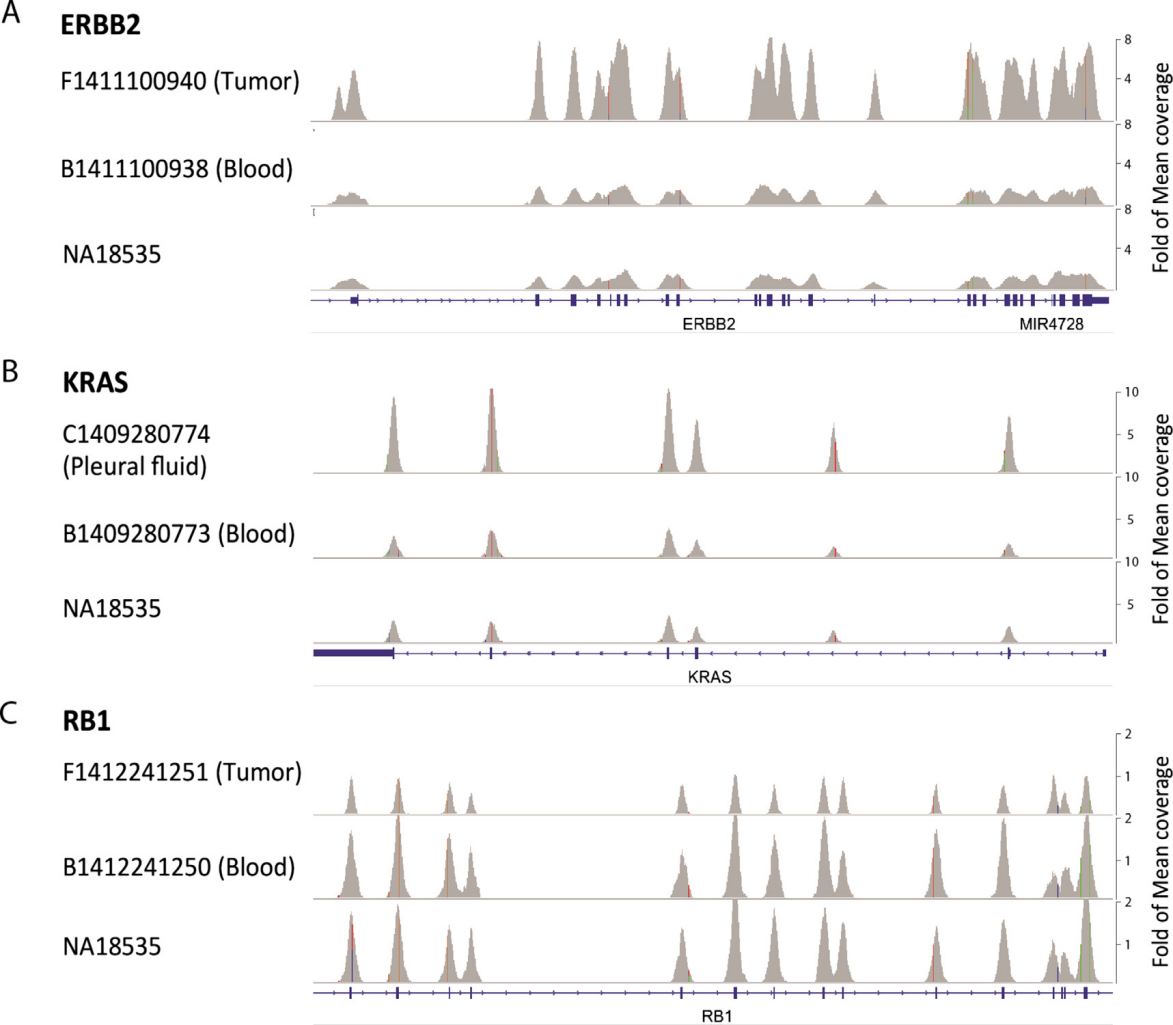
Examples of copy number variations(CNVs) identified by next‐generation sequencing (NGS). Sequence alignment data of ERBB2 (A), KRAS (B) and RB1 (C) was viewed by Integrative Genomics Viewer (IGV). Matched blood sample and NA18535 DNA was served as control. RefSeq Gene track was shown at the bottom. Due to the difference in coverage depth, samples are presented at the same fold of mean coverage depth for all matching and normal control samples as labeled on the right.

**Figure 5:**
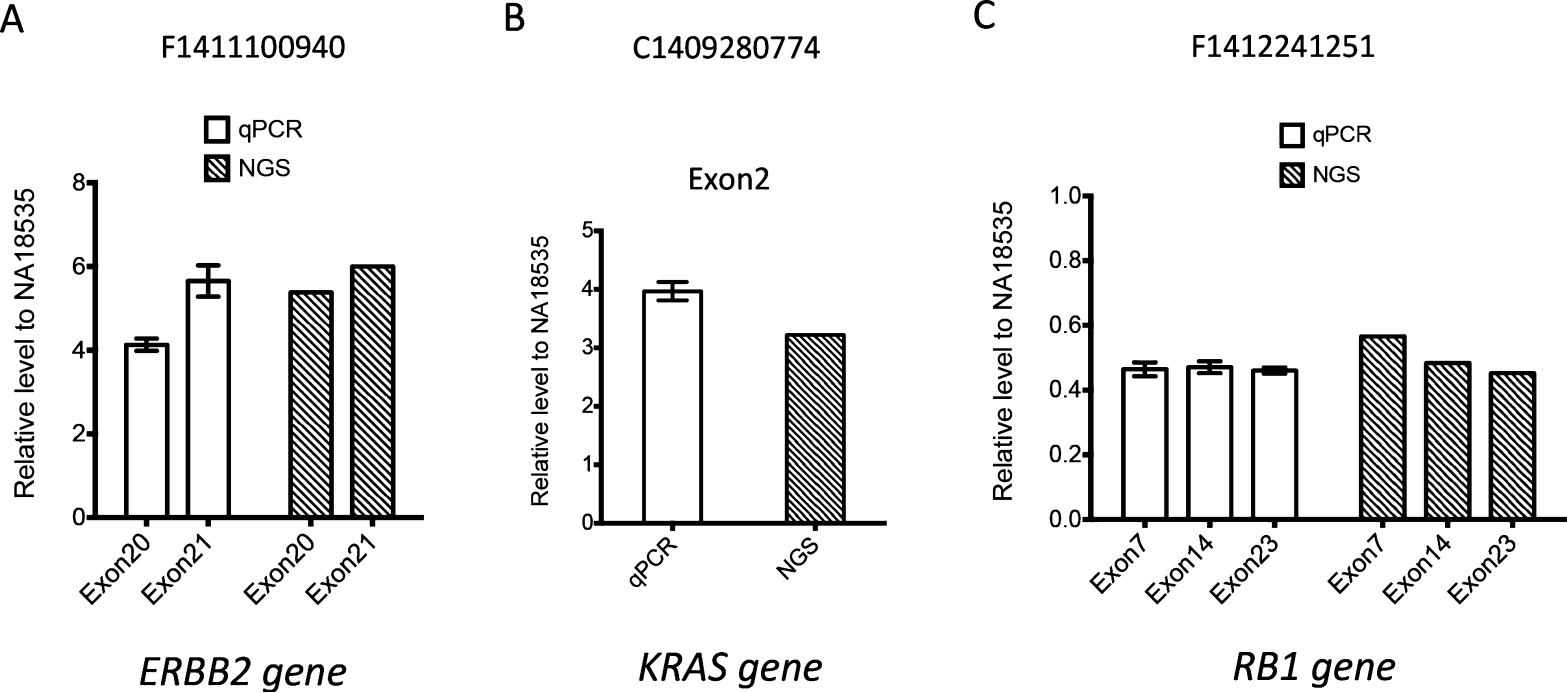
Validation of gene amplification by quantitative PCR (qPCR). Relative levels of amplified exons of three representative genes ERBB2 (A), KRAS (B) and RB1 (C) identified by next‐generation sequencing (NGS) were detected by qPCR, which was further normalized by the relative level of reference *ZNF80* gene region. The fold change for certain exon was calculated by normalizing to its relative level in normal control sample NA18535. Each value represents the mean ± SEM of three independent experiments for qPCR results. Copy number change detected by NGS was also plotted together with qPCR data on the right.

CNVs were detected using ADTEx (Amarasinghe et al. 2013) (See Materials and Methods). The examples for copy number gain (*ERBB2* and *KRAS*) and loss (*RB1*) detected by NGS were shown in Figure 4. To validate the accuracy of CNVs detection, real‐time qPCR was used for targeted exons of test genes and *ZNF80* gene (reference gene) (Fig. 5). Normal human hapmap genomic DNA NA18535 was used as normal control sample. The results showed that the copy number change identified by NGS was validated by the qPCR results.

### Clinical implications for target therapy

From our NGS‐based cancer genes genomic testing, functional genetic mutations were detected in these patient samples (Table S4). The detected actionable mutations along with the direct clinical implications were shown in the Table 3. Here, we found, in *ERBB2* gene (*EGFR* family member, also known as *HER2*), the c.2329G>T missense mutation caused amino acid substitution (p.Val777Leu) which resulted in excessive activation of downstream signaling pathways in breast cancer, lung cancer, and other tumors (Greulich et al. 2012; Bose et al. 2013). Tumor cells with activating mutations in *ERBB2* gene respond well to ERBB2 inhibitors such as Trastuzumab (Table 3) (Bose et al. 2013).

**Table 3:** Clinical implication of mutation identified

Mutation identified	Drug available	Drug resistance	Biological function
*ERBB2* c.2329G>T (p.Val777Leu)	Trastuzumab Lapatinib Neratinib	Unknown	Promote tumor cell proliferation, invasion and metastasis
*TP53* c.472C>T(p.Gln158*)	n/a	Platinum‐resistance	Promote tumor development
*ERCC5* c.3310G>C (p.Asp1104His)	n/a	Platinum toxicity	Increase tumor susceptibility, such as non‐small‐cell lung cancer
*EGFR* c.2573T>G (p.Leu858Arg)	EGFR‐TKIs	n/a	Promote tumor cell proliferation, invasion and metastasis
*EGFR* c.2369C>T (p.Thr790Met)	Afatinib Lapatinib	First‐generation EGFR‐TKIs resistance	Reduces the potency of ATP‐competitive kinase inhibitor
*EGFR* c.2235_2249del (p.Lys745_Ala750del)	EGFR‐TKIs	n/a	Promote tumor cell proliferation, invasion and metastasis
*PIK3CA* c.1636C>A (p.Gln546Lys)	Everolimus Temsirolimus	Reduces the sensitivity to EGFR and ERBB2 target drugs	Promote tumor cell proliferation, invasion and metastasis
*ERBB2* amplification	Trastuzumab Pertuzumab Lapatinib Afatinib	n/a	Promote tumor cell proliferation, invasion and metastasis
*KRAS* amplification	n/a	EGFR‐TKIs resistance	Promote tumor development

The *EGFR* is another *EGFR* family member involved in the pathogenesis and progression of different carcinomas (Normanno et al. 2006). In our study, we detected exon 19 deletion or exon 21 L858R point mutation in a NSCLC patient. These mutations increased EGFR kinase activity and resulted in hyperactivation of downstream prosurvival signaling pathways (Ladanyi and Pao 2008). In the treatment of mutated *EGFR* lung cancer, the first generation of tyrosine kinase inhibitors (TKIs) is commonly used, however, the efficacy of TKIs is limited due to the emergence of drug‐resistant secondary mutation T790M (Table 3), which increases ATP affinity at the ATP‐binding pocket and confers drug resistance (Yun et al. 2008). Thus, the irreversible inhibitors, such as Afatinib and Lapatinib, are capable of overcoming this resistance through covalent binding.

Inactivation of *TP53* occurs in more than half (~60%) of the cancers, and is a sign of poor prognosis in many types of cancer (Olivier et al. 2010; Muller and Vousden 2013). We found that c.472C>T mutation hotspot in *TP53* caused premature termination of codon formation, resulting in a truncated p53 that promoted tumor development and drug resistance to platinum treatment (Brachova et al. 2015). Additionally, we detected c.3310G>C polymorphism (rs17655) in *ERCC5* (also known as *XPG*), which is involved in platinum‐based drug‐induced DNA damage repair (Saldivar et al. 2007). The mutated *ERCC5* (p.Asp1140His, Table 3) lost the ability to repair DNA damage caused by platinum‐based drug treatment, resulting in increased toxicity (Zhu et al. 2012; He et al. 2013). These results indicate that the actionable mutations detected by our NGS protocol are capable of providing more accurate information for treating cancer patients, in contrast this valuable information may be missed in the regular Sanger sequencing, particularly when the sample contains limited tumor cells or the gene shows low‐mutating frequency.

## Discussion

Formalin fixation and paraffin embedding (FFPE) is a standard method for long‐term preservation of most archived pathological specimens. FFPE tissue is an excellent source of DNA, but its extraction remains a challenge. Formaldehyde, the effective component of formalin, leads to the generation of cross‐linking between nucleic acids and proteins (Gilbert et al. 2007), and causes nucleic acids to fragment because of fixation process conditions, such as extremely low pH (<1). Cross‐linking not only causes problems in DNA extraction, but blocks PCR amplification. Considerable effort has been made to optimize methods for extracting high‐quality DNA from FFPE samples. Shi et al. (2004) suggested that heating FFPE samples at a higher temperature in 0.1 mol/L NaOH solution highly increased the efficiency of DNA extraction. In our protocol, we have adopted the step for heating protein K digested FFPE DNA samples at 90° for 1 h, which greatly improved the productivity of FFPE derived DNA.

Poor quality and low amount of DNA also greatly influences the library preparation efficiency. Using Illumina TruSeq DNA PCR‐Free Sample Preparation Kit, sequencing library could be successfully generated from as little as 25 ng genomic DNA. Limited PCR amplification cycles were applied to low DNA input samples in order to increase the amount of library for later enrichment with minimum increase on PCR duplicates using NEB Next High‐Fidelity PCR master mix, which is specially optimized for the robust, high‐fidelity amplification of NGS libraries even with GC‐rich amplicons. On the other hand, libraries generated from poor quality DNA tented to have low PCR efficiency. Therefore, comprehensive pooling guideline needed to be applied in order to compensate the different PCR efficiency for each sample during postcapture PCR amplification.

The percentage of tumor content greatly influences the sensitivity of mutation identification, especially by traditional Sanger sequencing method. In our study, an *EGFR* p.Thr790Met mutant with 13% frequency was detected by NGS analysis in one patient's body fluid, which could be barely detected by Sanger validation. It is very easy to be missed when doing de novo testing for this mutant using Sanger method since it is very close to the detecting limit. As a result, important drug‐resistant information can be missed, and dramatically influences patient treatment decision. Therefore, our NGS‐based cancer genes genomic testing is a sensitive and efficient method to detect low abundant mutations. On the other hand, traditional clinical testing can only detect very limited markers, which will lose the whole picture of cancer genome. For example, in one patient with *EGFR* activating mutation, we also detected *KRAS* amplification, which will not be tested at the same time by traditional clinical testing, but will cause EGFR‐TKI resistance. As many more cancer‐related and therapeutically relevant genes have been discovered, additional target genes need to be added to our current panel. To this end, IDT xGEN lockdown probes greatly offered us the flexibility of expanding the current panel with additional customized DNA probes.

In summary, we have developed and validated an NGS‐based cancer genomic diagnosis test targeting 115 cancer‐related and therapeutically relevant genes on multiple types of cancer and specimens including difficult FFPE DNA samples. Using our self‐developed NGS data bioinformatics analysis pipeline, we were able to detect base substitutions, indels and gene CNVs. Our test possesses high analytical sensitivity, specificity and accuracy, supported a direct clinical use for this method to guide targeted therapy.

## Conflict of Interest

Xue Wu, Chao Zhang, Xiaofang Huang, Zhili Chang, and Yang Shao are the employees or shareholders of Geneseeq Technology Inc.

## Supporting information


**Table S1.** Gene targeted in hybridization capture.
**Table S2.** qPCR primer sequences.
**Table S3.** Summary of SNVs/Indels validation by Sanger sequencing.
**Table S4.** Mutation identified in all samples.

**Figure S1.** Integrity of genomic DNA extract from blood and FFPE samples.

**Figure S2.** Size distribution of sequencing library.
