# Supplementary material for: Capture‐based next‐generation sequencing reveals multiple actionable mutations in cancer patients failed in traditional testing
Source: Mol Genet Genomic Med. 2016 Jan 10;4(3):262–72. doi: 10.1002/mgg3.201 (PMC4867560; doi:10.1002/mgg3.201)
Supplement: Supplementary file 1 — Table S1. Gene targeted in hybridization capture. Table S2. qPCR primer sequences. Table S3. Summary of SNVs/Indels validation by Sanger sequencing. Table S4. Mutation identified in all samples. [file MGG3-4-262-s001.docx]

**S1 Table** Gene targeted in hybridization capture

| AIP | CDKN2B | FANCA | KRAS | PMS1 | SMARCB1 |
| --- | --- | --- | --- | --- | --- |
| AKT1 | CEBPA | FANCB | MAP2K4 | PMS2 | SOX2 |
| ALK | CEP57 | FANCC | MAX | PRF1 | STK11 |
| APC | CHEK2 | FANCD2 | MDM2 | PRKAR1A | SUFU |
| ARID1A | CTNNB1 | FANCE | MEN1 | PTCH1 | TGFBR2 |
| ATM | CYLD | FANCF | MET | PTEN | TMEM127 |
| BAP1 | DDB2 | FANCG | MLH1 | RAD51C | TP53 |
| BCL2 | DICER1 | FANCI | MSH2 | RAD51D | TSC1 |
| BLM | DIS3L2 | FANCL | MSH6 | RB1 | TSC2 |
| BMPR1A | DLG2 | FANCM | MUTYH | RECQL4 | VEGFA |
| BRAF | EGFR | FGFR1 | NBN | RET | VHL |
| BRCA1 | EPCAM | FGFR3 | NF1 | RHBDF2 | WRN |
| BRCA2 | ERBB2 | FH | NF2 | RUNX1 | WT1 |
| BRIP1 | ERCC2 | FLCN | NOTCH1 | SBDS | XPA |
| BUB1B | ERCC3 | GATA2 | NRAS | SDHAF2 | XPC |
| CDC73 | ERCC4 | GPC3 | NSD1 | SDHB |  |
| CDH1 | ERCC5 | HNF1A | PALB2 | SDHC |  |
| CDK4 | EXT1 | HRAS | PDGFRA | SDHD |  |
| CDKN1C | EXT2 | JAK2 | PHOX2B | SLX4 |  |
| CDKN2A | EZH2 | KIT | PIK3CA | SMAD4 |  |

**S2 Table** qPCR primer sequences

| qPCR Primer | Forward primer | Reverse primer |
| --- | --- | --- |
| HG-ERBB2-E20-F/R | ATACGTGATGGCTGGTGTGG | GGTTTTCCCGGACATGGTCT |
| HG-ERBB2-E21-F/R | CTCGTACACAGGGACTTGGC | TCTGTCTCGTCAATGTCCAGC |
| HG-RB1-E7-F/R | ATGATCTGGTGATTTCATTTCAG | TGAGCAACATGGGAGGTGAG |
| HG-RB1-E14-F/R | GTGATTTTCTAAAATAGCAGGCT | ATACAAGCGAACTCCAAGTTTG |
| HG-RB1-E23-F/R | ACCTCACATTCCTCGAAGCC | GTTGGTGTTGGCAGACCTTC |
| HG-KRAS-F/R | AGAATGGTCCTGCACCAGTAA | AGGCCTGCTGAAAATGACTGA |
| HG-ZNF80-F/R | CTGTGACCTGCAGCTCATCCT | TAAGTTCTCTGACGTTGACTGATGTG |

**S3 Table** Summary of SNVs/Indels validation by Sanger sequencing

| Sample ID | Sample type | Gene | Mutations detected by NGS(cDNA) | Sanger validation confirmed |
| --- | --- | --- | --- | --- |
| F1311260008 | FFPE | MSH2 | c.2055A>G | Yes |
| B1312160009 | Blood | KIT | c.910A>G | Yes |
| B1312160009 | Blood | NOTCH1 | c.1750G >A | Yes |
| F1312230017 | FFPE | CDKN2A | c.28G>T | Yes |
| F1312230017 | FFPE | TP53 | c.115G>T | Yes |
| F1401170002 | FFPE | ALK | c.4381A> G | Yes |
| F1401170002 | FFPE | ERBB2 | c.2329G>T | Yes |
| F1401170002 | FFPE | TP53 | c.98C>G | Yes |
| F1401170004 | FFPE | APC | c.7504G >A | Yes |
| F1401170004 | FFPE | BRCA2 | c.8131G>A | Yes |
| F1401170004 | FFPE | TP53 | c.472C>T | Yes |
| F1402240017 | FFPE | BRCA2 | c.10234A>G | Yes |
| F1402240017 | FFPE | ERBB2 | c.3508C>G | Yes |
| F1402240017 | FFPE | ERCC5 | c.3310G> C | Yes |
| F1402110008 | FFPE | ERBB2 | c.1963A>G | Yes |
| F1402110008 | FFPE | ERCC5 | c.3310G>C | Yes |
| C1409280774 | Fluid | EGFR | c.2573T>G | Yes |
| C1409280774 | Fluid | EGFR | c.2369C >T | Yes |
| C1409280774 | Fluid | KIT | c.1123A>C | Yes |
| F1410200833 | FFPE | EGFR | c.2369C>T | Yes |
| F1410200833 | FFPE | APC | c.4516_4521del | Yes |
| F1410200833 | FFPE | APC | c.4521_4522insCC | Yes |
| F1410200833 | FFPE | ARID1A | c.2775delA | Yes |
| F1410200833 | FFPE | BRCA1 | c.3124A>C | Yes |
| F1410200833 | FFPE | RECQL4 | c.448T>A | No |
| F1412241251 | FFPE | EGFR | c.2235_2249del | Yes |
| F1412241251 | FFPE | PIK3CA | c.1636C>A | Yes |
| F1412241251 | FFPE | TP53 | c.58C>T | Yes |

**S4 Table** Mutation identified in all samples

| Patient ID | Sample ID | Mutation identified |
| --- | --- | --- |
| 1 | F1311260008 | MSH2: Ile685Met  PTCH1: Ser676Gly |
| 3 | F1312230017 | CDKN2A: Glu10*  TP53: Glu39*  WT1: Lys247* and 247_249del |
| 4 | F1401170002 | ERBB2: amplification (2.5X) and Val777Leu  ALK: Ile1461Val  RUNX1: Asp332Asn  NSD1: Val345Leu and Ser457Pro  TP53: Pro33Arg |
| 5 | F1401170004 | APC: Gly2502Ser  BRCA2: Ala2711Thr  DICER1: Leu505Phe  PMS2: Arg20Gln and Lys541Glu  TP53: Pro72Arg and Gln158* |
| 6 | F1402240017 | BRCA2: Ile3412Val  ERCC5: Asp1104His  PMS2: Thr485Lys and Lys541Glu  TGFBR2: Glu150fs |
| 7 | F1410200833 | EGFR: Thr790Met and 745_750del in exon19  APC: 1523_1524del and Met1525Thr  ARID1A: Gly925fs  BRCA1:Ser1042Arg  ERBB2: Ile625Val  TP53: Pro33Arg |
| 8 | C1409280774 | EGFR: Leu858Arg and Thr790Met  BRCA2: Asn372His  KIT: Ser375Arg  KRAS: amplification (3.2X)  TP53: Arg26Leu |
| 9 | F1411100940 | ERBB2: amplification (4.2X) and I625V  NOTCH1: amplification (1.7X) |
| 10 | F1412241251 | EGFR: 745_750del in exon19  ERBB2: amplification (2X)  PIK3CA: Gln546Lys  ERCC2: amplification (2.2X)  RB1: one copy loss  TP53: Pro72Arg and His179Tyr |
