# Supplementary figures and images for: Capture‐based next‐generation sequencing reveals multiple actionable mutations in cancer patients failed in traditional testing
Source: Mol Genet Genomic Med. 2016 Jan 10;4(3):262–72. doi: 10.1002/mgg3.201 (PMC4867560; doi:10.1002/mgg3.201)

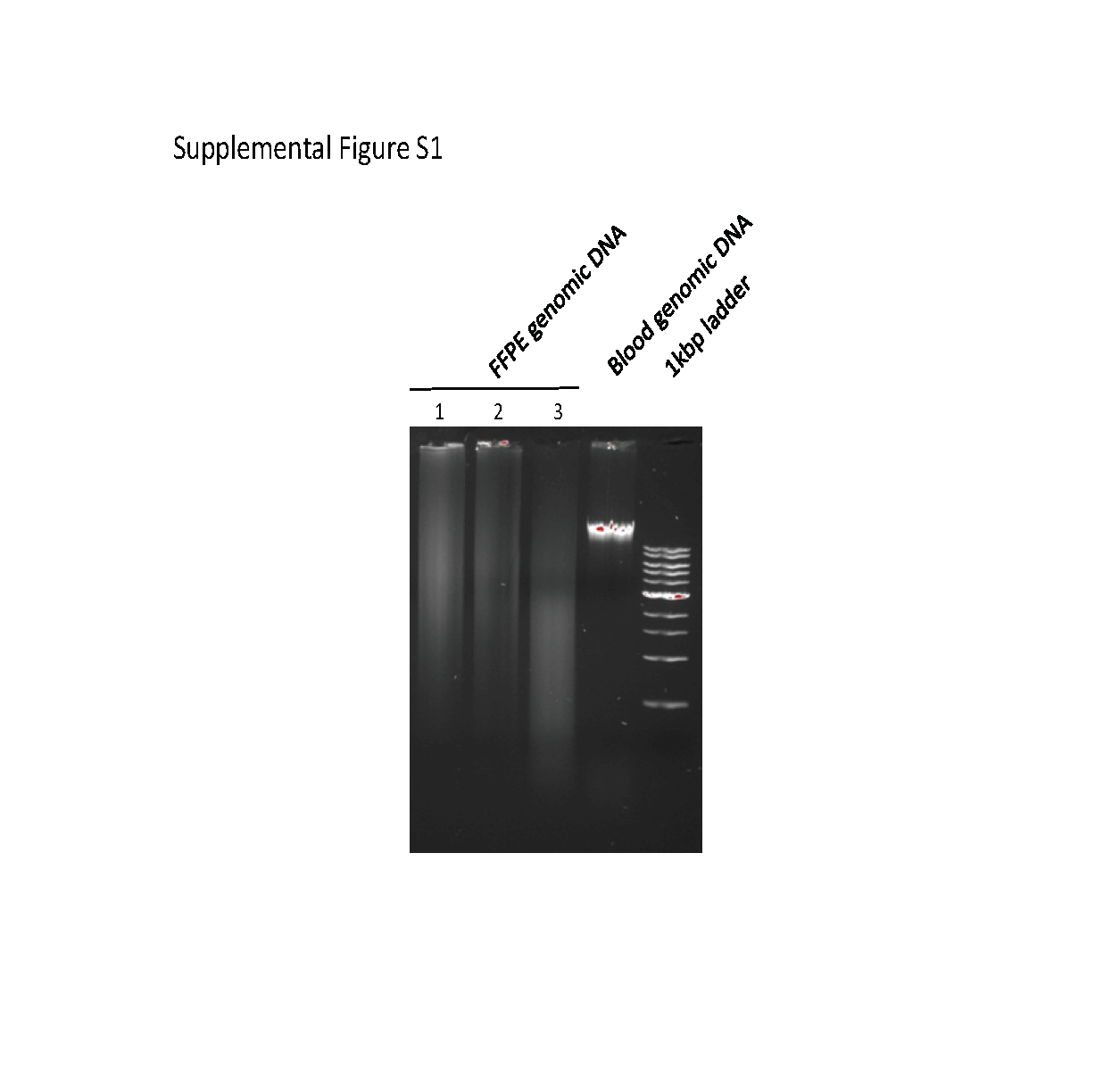

Supplement: Supplementary file 2 — Figure S1. Integrity of genomic DNA extract from blood and FFPE samples. [file MGG3-4-262-s002.tif]

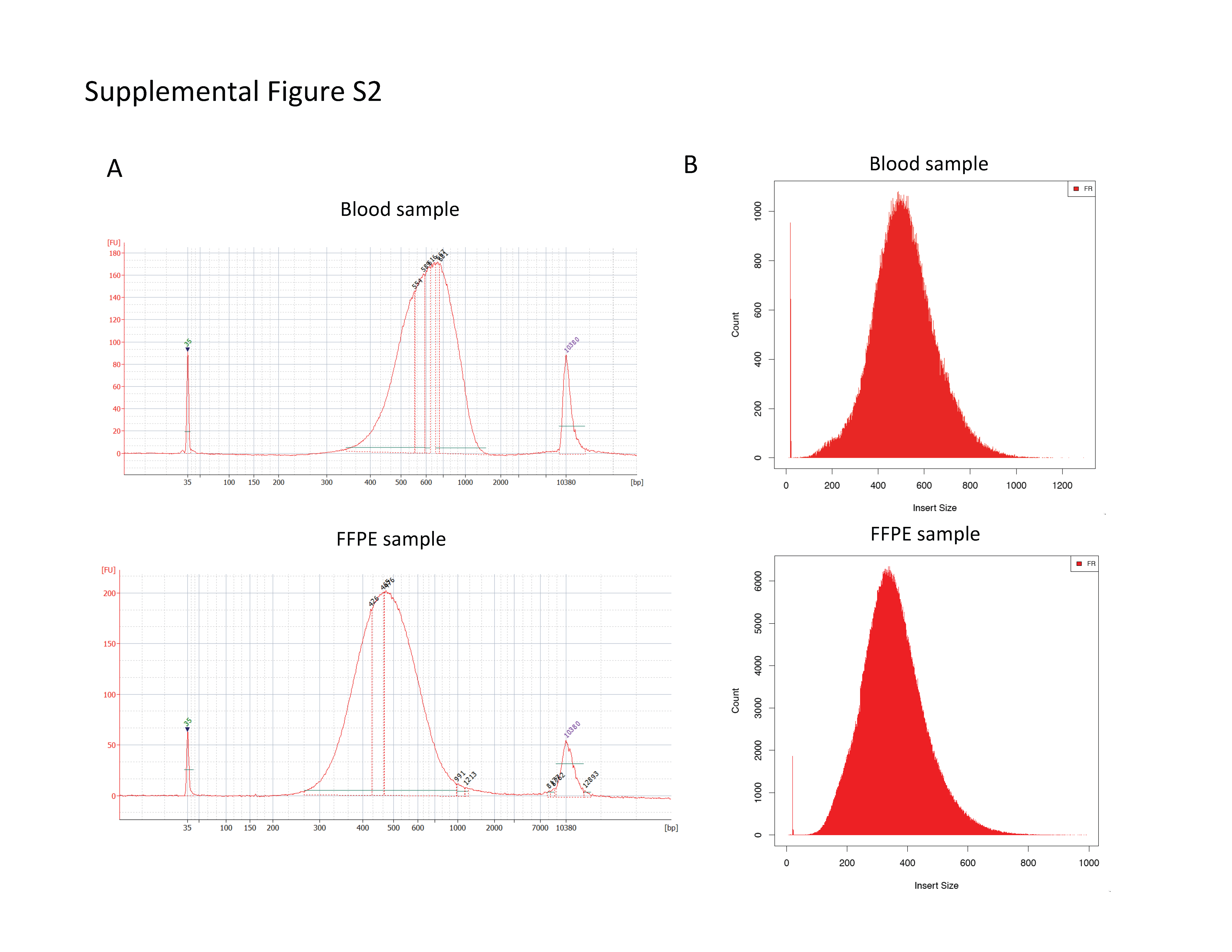

Supplement: Supplementary file 3 — Figure S2. Size distribution of sequencing library. [file MGG3-4-262-s003.tif]
