# Supplementary material for: Capture‐based next‐generation sequencing reveals multiple actionable mutations in cancer patients failed in traditional testing
Source: Mol Genet Genomic Med. 2016 Jan 10;4(3):262–72. doi: 10.1002/mgg3.201 (PMC4867560; doi:10.1002/mgg3.201)
Supplement: Supplementary file 4 [file MGG3-4-262-s004.docx]

**Titles and legends to figures**

**Supplemental Figure 1**. Integrity of genomic DNA extract from blood and FFPE samples.

10ng of genomic DNA was separated on 1% agarose gel. Genomic DNA extracted from blood samples kept intact, while those extracted from FFPE samples had different degrees of fragmentation.

**Supplemental Figure 2** Size distribution of sequencing library

(A) Agilent Bioanalyzer trace of typical blood (top) and FFPE (bottom) sequencing library samples. (B) Insert size distribution of typical blood (top) and FFPE (bottom) sequencing samples obtained from NGS data.
